# Preoperative and intraoperative assessment of myometrial invasion in patients with FIGO stage I non-endometrioid endometrial carcinoma—a large-scale, multi-center, and retrospective study

**DOI:** 10.1186/s13000-023-01294-z

**Published:** 2023-01-25

**Authors:** Xiaohang Yang, Jingjing Yin, Yu Fu, Yuanming Shen, Chuyao Zhang, Shuzhong Yao, Congjian Xu, Min Xia, Ge Lou, Jihong Liu, Bei Lin, Jianliu Wang, Weidong Zhao, Jieqing Zhang, Wenjun Cheng, Hongyan Guo, Ruixia Guo, Fengxia Xue, Xipeng Wang, Lili Han, Xiaomao Li, Ping Zhang, Jianguo Zhao, Wenting Li, Yingyu Dou, Zizhuo Wang, Jingbo Liu, Kezhen Li, Gang Chen, Chaoyang Sun, Pengming Sun, Weiguo Lu, Qin Yao

**Affiliations:** 1 Cancer Biology Research Center (Key Laboratory of the Ministry of Education), Tongji Hospital, Tongji Medical College, Huazhong University of Science and Technology, Wuhan, Hubei 430000 China; 2 Department of Gynecology and Obstetrics, Tongji Hospital, Tongji Medical College, Huazhong University of Science and Technology, Wuhan, Hubei 430000 China; 3 Women’s Hospital, School of Medicine, Zhejiang University, Hangzhou, 310000 China; 4 Department of Gynecologic Oncology, Sun Yat-Sen University Cancer Center, 651 Dongfeng E Rd, Guangzhou, 510060 China; 5 Department of Obstetrics and Gynecology, The First Affiliated Hospital of Sun Yat-Sen University, No 58. Zhong Shan ER Lu, Guangzhou, 510080 China; 6 Department of Gynecology, Obstetrics and Gynecology Hospital of Fudan University, Shanghai, China; 7 Department of Gynecology and Obstetrics, The Affiliated Yantai Yuhuangding Hospital of Qingdao University, NO 20 Yuhuangding East Road, Yantai, Shandong 264000 China; 8 Department of Gynecology Oncology, Harbin Medical University Cancer Hospital, Harbin, 150086 China; 9 Department of Obstetrics and Gynecology, Shengjing Hospital Affiliated to China Medical University, No. 36, Sanhao Street, Heping District, Shenyang, Liaoning 110004 China; 10 Peking University People’s Hospital, Beijing, 100044 China; 11 The First Affiliated Hospital of USTC, Division of Life Sciences and Medicine, University of Science and Technology of China, Hefei, Anhui 230001 China; 12 Department of Gynecologic Oncology, Guangxi Medical University Cancer Hospital, 71 Hedi Road, Nanning, Guangxi 530021 China; 13 The First Affiliated Hospital of Nanjing Medical University, 300 Guangzhou Road, Gulou District, Nanjing, Jiangsu 210029 China; 14 The Third Hospital of Peking University, 49 North Garden Rd., Haidian District, Beijing, China; 15 Department of Gynecology and Obstetrics, The First Affiliated Hospital of Zhengzhou University, No.1, Jianshe East Road, Zhengzhou, 450052 China; 16 Department of Gynecology and Obstetrics, Tianjin Medical University General Hospital, 154 Anshan Dao, Heping District, Tianjin, 300052 China; 17 Department of Gynecology and Obstetrics, XinHua Hospital, Shanghai JiaoTong University School of Medicine, Shanghai, 200092 China; 18 Department of Gynecology, People’s Hospital of Xinjiang Uygur Autonomous Region, No. 91 Tianchi Street, Tianshan District, Urumqi, 830001 China; 19 Department of Gynecology and Obstetrics, The Third Affiliated Hospital, Sun Yat-Sen University, No. 600 Tianhe Road, Tianhe District, Guangzhou, 510630 China; 20 Department of Gynecology, The Second Hospital of Shandong University, 247 Bei Yuan Street, Jinan, Shandong 250033 China; 21 Department of Gynecologic Oncology, Tianjin Central Hospital of Gynecology and Obstetrics, Affiliated Hospital of Nankai University, No. 156, Sanma Road, Nankai District, Tianjin, 300100 China; 22 Tianjin Clinical Research Center for Gynecology and Obstetrics, Affiliated Hospital of Nankai University, No. 156, Sanma Road, Nankai District, Tianjin, 300100 China; 23 Branch of National Clinical Research Center for Gynecology and Obstetrics, Affiliated Hospital of Nankai University, No. 156, Sanma Road, Nankai District, Tianjin, 300100 China; 24 Fujian Provincial Women & Children’s Hospital, Fujian Provincial Maternity & Children Health Hospital, Fujian Medical University, Fuzhou, Fujian 350000 China; 25 Department of Obstetrics and Gynecology, The Affiliated Hospital of Qingdao University, 16 Jiangsu Road, Qingdao, Shandong 266003 China

**Keywords:** Non-endometrioid endometrial carcinoma, Frozen sections, Myometrial invasion, Lymph node dissection, Retrospective studies

## Abstract

**Introduction:**

Myometrial invasion is a prognostic factor for lymph node metastases and decreased survival in non-endometrioid endometrial carcinoma patients. Herein, we explored the mode of myometrial invasion diagnosis in FIGO stage I non-endometrioid carcinoma and evaluated the differences in diagnostic efficiency among intraoperative frozen section (IFS), intraoperative gross examination (IGE), magnetic resonance imaging (MRI), and computed tomography (CT) in clinical practice. Finally, we suggested which test should be routinely performed.

**Method:**

This was a historical cohort study nationwide with 30 centers in China between January 2000 and December 2019. Clinical data, including age, histology, method of myometrial invasion evaluation (MRI, CT, IGE, and IFS), and final diagnosis of postoperative paraffin sections, were collected from 490 non-endometrioid endometrial carcinoma (serous, clear cell, undifferentiated, mixed carcinoma, and carcinosarcoma) women in FIGO stage I.

**Results:**

Among the 490 patients, 89.59% presented myometrial invasion. The methods reported for myometrial invasion assessment were IFS in 23.47%, IGE in 69.59%, MRI in 37.96%, and CT in 10.20% of cases. The highest concordance was detected between IFS and postoperative paraffin sections (Kappa = 0.631, accuracy = 93.04%), followed by IGE (Kappa = 0.303, accuracy = 82.40%), MRI (Kappa = 0.131, accuracy = 69.35%), and CT (Kappa = 0.118, accuracy = 50.00%). A stable diagnostic agreement between IFS and the final results was also found through the years (2000–2012: Kappa = 0.776; 2013–2014: Kappa = 0.625; 2015–2016: Kappa = 0.545; 2017–2019: Kappa = 0.652).

**Conclusion:**

In China, the assessment of myometrial invasion in non-endometrioid endometrial carcinoma is often performed via IGE, but the reliability is relatively low in contrast to IFS. In clinical practice, IFS is a reliable method that can help accurately assess myometrial invasion and intraoperative decision-making (lymph node dissection or not). Hence, it should be routinely performed in non-endometrioid endometrial carcinoma patients.

**Supplementary Information:**

The online version contains supplementary material available at 10.1186/s13000-023-01294-z.

## Introduction

Endometrial carcinoma is one of the most common malignant tumors of the female reproductive tract in developed countries, accounting for almost 5% of women's cancer worldwide [1]. Although most endometrioid adenocarcinoma patients have favorable prognoses, non-endometrioid endometrial carcinoma can be more aggressive, along with a higher risk for lymphatic involvement [2]. The surgery area is an important prognostic factor and can provide information about lymph node metastases, which is vital to accurately determining the stage and deciding the postoperative therapy. However, lymphadenectomy is always recommended for women with non-endometrioid endometrial carcinoma, and myometrial invasion (MI) assessment has not been mandatory in previous clinical decisions [3].

According to the latest ESGO 2021 guidelines [4], stage I non-endometrioid (serous, clear cell, undifferentiated carcinoma, carcinosarcoma, and mixed) without MI (< 50% or ≥ 50% of the myometrium) patients are classified into an intermediate-risk group, and stage I non-endometrioid with MI, and with no residual disease is classified into a high-risk group. Surgical lymph node staging should be performed in women with high-intermediate/ high-risk endometrial carcinoma. In contrast, for women with low/intermediate risk, there is no need to perform systematic lymphadenectomy to avoid risks and complications such as lymphoedema or lymph cyst formation. Hence, the presence of MI or not plays a key role in intraoperative decisions of non-endometrioid endometrial carcinoma patients.

Preoperative magnetic resonance imaging (MRI) and transvaginal sonography (TVS) have been widely used to assess MI [5]. However, some limitations undermine their reliability, including insufficient reader experience, interobserver variability, and the technical level of the operator [6–8]. Intraoperative gross evaluation (IGE) is another option available, but research has shown that 25% of cases can be undertreated. This underestimation might result from the different levels of experience, poor histological differentiation, and multiple foci, which can confuse the evaluation of tumor invasion [9]. For better detection, an intraoperative frozen section (IFS) is a choice in some gynecological centers, but current studies in endometrial carcinoma have found conflicting insights on IFS accuracy [10]. Some studies have found that IFS is an accurate and referable implement for guiding intraoperative decision-making. For example, the percentage of cases receiving sub-optimal surgical management due to IFS errors was as low as 5.3% [11]. In a prospective study with 784 women from Mayo Clinic, the rate amounted to 1.3%, respectively [12]. In contrast, several papers have demonstrated a poor correlation between IFS and final diagnosis, possibly because IFS has some limitations, such as block selection error, artifacts, lack of extensive sampling, and inadequate experience of expert pathologists [13–17]. Preoperative CT, MRI, IGE, and IFS analysis are widely used to assess deep MI (≥ 50% of the myometrium) in endometrioid adenocarcinoma. Various studies have identified their diagnostic efficacy and prioritized them. However, there is no consensus regarding which pre/intraoperative diagnostic method for evaluating MI should be preferred in non-endometrioid endometrial carcinoma patients.

Therefore, in the present study, we aimed to explore the pattern of different diagnostic methods for assessing MI in non-endometrioid endometrial carcinoma patients using data from 30 centers in China between January 2000 and December 2019. We also evaluated the methods' sensitivity, specificity, positive and negative predictive values, accuracy, and Kappa value. This was the first study to evaluate the diagnostic efficacy of various methods for MI in stage I non-endometrioid endometrial carcinoma in the largest historical cohort in China over the past 20 years.

## Materials and methods

### Patient and public involvement

To collect unique data on non-endometrioid endometrial carcinoma in China, we generated a database with 30 academic centers from different Chinese regions in 2018 and retrospectively collected data on women with discharge diagnosis of primary endometrial carcinoma from January 1, 2000, to December 31, 2019. This study was approved by Institutional Review Boards in all centers. We also generated an electronic database for data transfer and collection.

### Study design and quality assessment on diagnostic accuracy

**Fig. 1:**
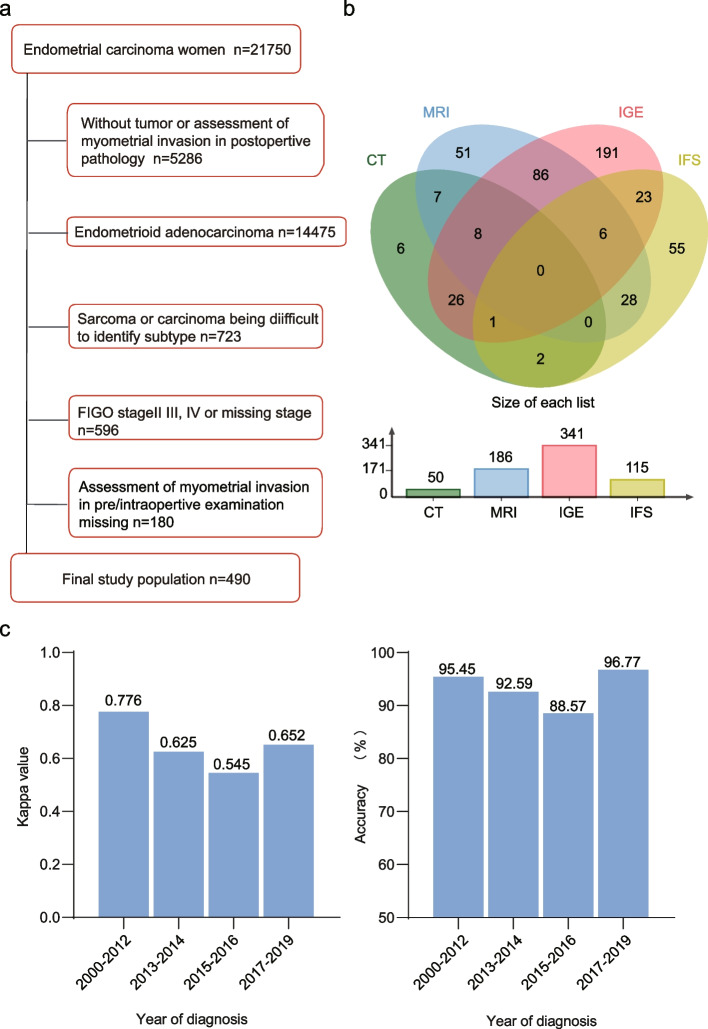
**a** Flow of participants, using a diagram; **b** Grouping of the final study population through the Wayne chart way (preoperative CT, preoperative MRI, intraoperative gross examination, and intraoperative frozen section); **c** The Kappa value and accuracy of intraoperative frozen section compared with the final histopathology by year of diagnosis

We consulted the Revised Tool for the Quality Assessment of Diagnostic Accuracy Studies (QUADAS-2) and established inclusion and exclusion criteria to eliminate selection bias [18]. In the endometrial carcinoma database with 21,750 cases, we first excluded 5286 cases without tumor or MI assessment in postoperative pathology. Then, we excluded 14,475 endometrioid adenocarcinoma cases and 723 sarcoma or cases that were difficult to diagnose. In the remaining 1266 participants with non-endometrioid endometrial carcinoma (serous, clear cell, undifferentiated, mixed carcinoma, and carcinosarcoma), 670 clinical-stage I patients were included. After ruling out 180 cases without MI assessment in pre/intraoperative examination, 490 women who underwent pre/intraoperative MI assessment were included for subsequent analysis (Fig. 1a). Some women underwent multiple examinations to assess MI (Fig. 1b). We attempted to compare the diagnostic ability of pre/intraoperative procedures (MRI, CT, IGE, and IFS) to assess MI, considering postoperative paraffin section (PS) pathology after hysterectomy as the gold standard for statistical analyses. Although a retrospective study was performed, we still referenced the QUADAS-2, a methodological quality assessment tool for meta-analysis of diagnostic trials to eliminate selection bias (Supplementary Table 1).

### Clinical pathway and definition

Among the 490 cases finally included, all patients underwent preoperative biopsy (curettage or hysteroscopy) and were diagnosed with endometrial carcinoma or highly suspected endometrial carcinoma at admission. Patients were examined and treated according to the clinical pathway of endometrial carcinoma after admission. During the period of database establishment, we re-checked all data according to the standardized protocols of CT, MRI, IGE, IFS and postoperative PS in order to rule out diagnostic bias in different institutions. The details of diagnostic criteria generated between 2018 to 2020 were presented in Supplementary materials. All results for the MI diagnosis were no and yes (superficial: < 50%; deep: ≥ 50% of the myometrium), and at least two clinical professionals verified the results.

### Statistical analysis

We used SPSS 27.0 software (IBM SPSS Statistics for Windows Armonk, NY: IBM Corporation) for statistical analyses. Diagnostic efficacy was evaluated using accuracy, sensitivity, specificity, positive predictive values (PPV), and negative predictive values (NPV), with corresponding 95% confidence intervals (CIs). We tested the agreement between methods using the Kappa test and Cronbach's α—inter rate correlation. The definitions and applications of various statistical indicators are detailed in Table 1.

**Table 1:** Statistical analysis

Statistical method	Definition
Sensitivity	True positive rate, TPR = TP/ (TP + FN)
Specificity	True negative rate, TNR = TN/ (FP + TN)
PPV	Positive predictive value = TP/ (TP + FP)
NPV	Negative predictive value = TN/ (TN + FN)
Accuracy	= TP + TN/ (TP + FN + FP + TN)
Cohen’s Kappa	Consistency; unordered dichotomous variables (kappa ≤ 0, consistency less than chance; kappa ≤ 0.20, slight consistency; kappa = 0.21–0.40, fair consistency; kappa = 0.41–0.60, moderate consistency; kappa = 0.61–0.80, substantial consistency; kappa > 0.8, almost perfect consistency)
Cronbach’s α—inter rate correlation	Consistency; ordered classified variables (value > 0.90, high correlation; value = 0.8–0.9, acceptable correlation; value = 0.70–0.8, scale needs amending; value < 0.7, discard)

*TP* True positive, *TN* True negative, *FP* False positive, *FN* False negative

## Results

### Final study cohort

The baseline characteristics of non-endometrioid endometrial carcinoma women with MI assessments, including age, year at diagnosis, MI assessment method, histology grade, and World Health Organization (WHO) pathological classification, are presented in Table 2. The median age at diagnosis was 59.29 ± 9.59 years. Moreover, 403 cases were diagnosed after 2012 (2012–2013: 12.45%; 2014–2015: 30.82%; 2016–2017: 34.90%; 2018–2019:4.08%), and 87 cases were diagnosed between 2000–2011. The MI prevalence was 89.59% in the study population based on the final histopathology (Table 2). Among the 490 included women, 272 (55.51%) were diagnosed with serous carcinoma and 26.12% with clear cell carcinoma. Additionally, fewer than half of women were in low-risk grades (grade 1: 26.12%; grade 2: 20.82%).

**Table 2:** Clinical and pathological characteristics of the study population

Study population	(%) for cases with observed data
Age at diagnosis (year), median ± standard error	59.29 ± 9.59
MI	
No	51 (10.41%)
Yes	439 (89.59%)
Year of diagnosis	
2000–2007	21 (4.29%)
2008–2009	26 (5.31%)
2010–2011	40 (8.16%)
2012–2013	61 (12.45%)
2014–2015	151 (30.82%)
2016–2017	171 (34.90%)
2018–2019	20 (4.08%)
Evaluating method of MI	
CT	50 (10.20%)
MRI	186 (37.96%)
IGE	341 (69.59%)
IFS	115 (23.47%)
Pathological subtype	
Serous carcinoma	272 (55.51%)
Clear cell carcinoma	128 (26.12%)
Undifferentiated carcinoma	29 (5.92%)
Carcinosarcoma	37 (7.55%)
Mixed	24 (4.90%)
Grade	
Grade 1	128 (26.12%)
Grade 2	102 (20.82%)
Grade 3	168 (34.29%)
Undifferentiation	92 (18.78%)

*MI* Myometrial invasion, *IFS* Intraoperative frozen section, *MRI* Magnetic resonance imaging, *CT* Computerized tomography, *IGE* Intraoperative gross examination

### Evaluation on myometrial invasion

The final study population included 115 women with MI assessments by IFS, 341 by IGE, 186 by MRI, and 50 by CT (Table 3). The significantly high PPV (99.00%) and NPV (53.33%) of IFS supported avoiding unnecessary medical interventions. The IFS presented the highest sensitivity (93.40%), followed by IGE (84.24%), MRI (73.13%), and CT (45.65%). Additionally, CT (100.00%) and IFS (88.89%) had higher specificity than MRI (46.15%) and IGE (63.33%). The highest consistency was observed between IFS and the final pathology report (Cronbach's α = 0.780, Kappa = 0.631), demonstrating its high repeatability, followed by IGE (Cronbach's α = 0.483, Kappa = 0.303), MRI (Cronbach's α = 0.247, Kappa = 0.131), and CT (Cronbach's α = 0.350, Kappa = 0.118).

**Table 3:** Cronbach’s α—inter rate correlation, Kappa value, accuracy, sensitivity, specificity, PPV, NPV of the methods used for assessing myometrial invasion compared with the final paraffin-embedded pathology evaluation

MI	PS of postoperative pathology			Cronbach’s α	Kappa	Accuracy % (95% CI)	Sensitivity % (95% CI)	Specificity % (95% CI)	PPV % (95% CI)	NPV % (95% CI)
	**No**	**Yes**	**Total**							
**CT**										
No	4	25	29	0.350	0.118	50.00 (35.72- 64.28)	45.65 (31.18- 60.84)	100.00 (39.58–100.00)	100.00 (80.76–100.00)	13.79 (4.51- 32.57)
Yes	0	21	21							
Total	4	46	50							
**MRI**										
No	12	43	55	0.247	0.131	69.35 (62.11- 75.78)	73.13 (65.45- 79.68)	46.15 (27.14- 66.25)	89.31 (82.41- 93.82)	21.82 (12.25- 35.36)
Yes	14	117	131							
Total	26	160	186							
**IGE**										
No	19	49	68	0.483	0.303	82.40 (77.85- 86.21)	84.24 (79.60- 88.01)	63.33 (43.90- 79.45)	95.97 (92.70- 97.87)	27.94 (18.06- 40.34)
Yes	11	262	273							
Total	30	311	341							
**IFS**										
No	8	7	15	0.780	0.631	93.04 (86.33- 96.73)	93.40 (86.40- 97.08)	88.89 (50.67- 99.42)	99.00 (93.76- 99.95)	53.33 (27.42- 77.72)
Yes	1	99	100							
Total	9	106	115							

*IFS* Intraoperative frozen section, *MRI* Magnetic resonance imaging, *CT* Computerized tomography, *MI* Myometrial invasion, *IGE* Intraoperative gross examination, *PS* Paraffin section

We also calculated the Kappa value and accuracy for IFS divided by the year of diagnosis (Fig. 1c). We did not detect obvious variations in the diagnostic efficiency of IFS between 2000–2019 (2000–2012: Kappa = 0.776; 2013–2014: Kappa = 0.625; 2015–2016: Kappa = 0.545; 2017–2019: Kappa = 0.652). Overall, the IFS analysis maintained favorable effective functions by year of diagnosis.

## Discussion

According to the ESGO 2021 guidelines [4], for women with non-endometrioid tumors, MI assessment is also recommended to define prognostic risk groups (no MI: intermediate-risk group; MI: high-risk group). Surgical lymph node staging should be performed in high intermediate-risk/high-risk women. Sentinel lymph node (SLN) biopsy is an acceptable alternative to systematic lymphadenectomy when lesions are confined to the uterus in high/intermediate-high women. Recently, a study showed that the SLN concept was adopted by about 50% of surgeons and became widely used in 69 countries, especially in Europe and the USA [19]. However, few medical centers can perform SLN, particularly in developing countries. Moreover, accurately mapping SLN still has some challenges. Pathologic ultrastaging based on H&E staining allows accurate identification of SLN metastases but delays the final diagnosis due to tissue processing and staining [20, 21]. Although the delivery of results is fast, IFS might poorly sensitive to detect SLN metastases [22]. Besides, many emerging detection methods can quickly and accurately map SLN during surgery. Several studies have linked that one-step nucleic acid amplification (OSNA) is highly accurate for the intraoperative assessment of SLN in endometrial cancer [23]. However, OSNA has low reliabilities in some histotypes, such as carcinosarcoma, undifferentiated carcinoma, and dedifferentiated carcinoma [23]. Besides, over the years, there has been an argument about whether SLN mapping should be used in high-risk histologies (serous carcinoma, clear cell carcinoma, and carcinosarcoma) [24]. Accordingly, tools still need to select high-risk women for lymph node dissection in non-endometrioid endometrial carcinoma. Therefore, investigating which MI assessment methods are best is essential.

Our current results indicated that IFS is the first choice for evaluating MI in non-endometrioid endometrial carcinoma, followed by IGE, MRI, and CT. IFS correctly detected about 93.04% of MI assessments in preoperative non-endometrioid endometrial carcinoma patients. The NPVs were lower than 50% for IGE, MRI, and CT, showing that none of the methods is optimum for excluding non-MI patients, except IFS. Also, IFS presented the highest sensitivity (93.40%) to detect MI, whereas it was lower for IGE, MRI, and CT. We believe that this sensitivity might be relatively more important when estimating MI because more MI patients can be identified, staged, and treated accordingly. Furthermore, IFS had the lowest rate of false positives and the highest specificity. Hence, few patients will be unnecessarily staged with lymphadenectomies, resulting in an unnecessarily high rate of complications. Many previous evaluations only included correct results (sensitivity and specificity), but the false results, including false-positive and -negative cases, were not considered simultaneously [2, 25]. Herein, we used the Kappa consistency to check all results, which is more rigorous. We showed that IFS had the highest Kappa value, comprising a reliable method for MI assessment and intraoperative management of individuals with non-endometrioid endometrial carcinoma. However, sometimes, IFS examinations are performed by less experienced examiners. Therefore, more than two pathologists should perform the IFS for MI assessment.

In this nationwide cohort study of clinical MI assessment in stage I non-endometrioid endometrial carcinoma, IGE was the most common method reported, followed by MRI, IFS, and CT. Research has shown that it is challenging for IGE to determine MI, especially in low-grade tumors, as the invasion line can be heterogeneous with skip metastasis [26]. These results are consistent with our current findings that IGE consistency cannot reach a moderate level in non-endometrioid endometrial carcinoma. MRI has also been preoperatively employed as an alternative tool to evaluate the depth of MI [27]. The differences in medical imaging devices, radiological technology, and reading ability training in clinical practice are "defects of MRI." Although with good accuracy in some medical centers, MRI remains expensive and is not always available [28]. Meanwhile, the clinical application of IFS in endometrial carcinoma remains controversial. In non-endometrioid endometrial carcinoma, the endometrium can penetrate the basal layer without a clear boundary in standard anatomical structure, which is more likely to be misdiagnosed in MRI and IGE when the lesion is small or at the junction. As for IFS, tissue can generally be cut into thin slices of a few microns, and the tissue does not significantly shrink. The cell morphology does not change considerably without being treated with solvent or affected by the intense stimulation of reagent and temperature [29], comprising the "advantages of IFS in non-endometrioid endometrial carcinoma". Besides, some studies have supported the significant flaws of CT, which were also found here. Few studies have recommended CT for MI assessment, but it is widely used to evaluate extrauterine lesions and lymph node enlargement [30, 31].

The 2021 ESGO guidelines recommend that molecular classification should be encouraged in all endometrial carcinomas, including three immunohistochemical markers (p53, MSH6, and PMS2) and one molecular test (mutation analysis of the exonuclease domain of POLE) [4]. This surrogate marker approach to the molecular-based classification has been demonstrated to be prognostically informative in smaller studies with non-endometrioid tumors [4]. The integrated risk stratification system was encouraged to manage endometrial carcinomas in 2021 ESGO guidelines, although data regarding integrated molecular and histological prognostic factors remain scarce [32]. However, many studies have suggested that IFS is not encouraged for myometrial invasion assessment because of interference with adequate pathological processing [4]. Indeed, some of the proposed biomarkers require high-quality preanalytical treatment of surgical specimens, such as appropriate fixation conditions. So, there is a trade-off between the diagnostic priority in myometrial invasion assessments of IFS and the risk of interfering with pathological processing. Due to the limited application of the Proactive Molecular Risk Classifier for Endometrial Cancer (ProMisE) in China, evaluating the risk factors with unknown molecular classification for endometrial carcinoma is still an important step in the diagnosis and treatment of endometrial cancer. Assessing MI during surgery to guide the excision extent is a priority for some patients, and inexpensive and readily available IFS might be a better option.

Furthermore, the MI assessment from TVS was not included in our database. In the 30 included centers, the depth of MI was not requested in detail in the TVS reports of suspected non-endometrioid endometrial carcinoma women, and most clinicians were more dependent on the MI evaluation from MRI before operations. Not evaluating the MI parameter in TVS is another limitation and has resulted in data deletion while comparing the accuracy between methods.

In summary, we assessed the priorities of pre/intraoperative MI analysis methods in non-endometrioid endometrial carcinoma and included the largest group of women, comparing the accuracy of different approaches. A comprehensive evaluation was performed using the most suitable statistical method.

## Conclusion

In China, MI assessment in non-endometrioid endometrial carcinoma is usually performed via IGE. However, its sensitivity and specificity are lower than IFS in clinical practice. Hence, more non-endometrioid endometrial carcinoma patients are falsely classified and might not be primarily operated on correctly with lymphadenectomy as recommended, leading to under-staging or excessive surgical tissue removal.

We demonstrated that IFS is the most effective strategy to evaluate MI of non-endometrioid endometrial carcinoma and provides a more accurate reference than CT, MRI, and IGE. We recommend that women with intrauterine non-endometrioid endometrial carcinoma who need MI evaluation should be referred to clinics with a pathologist available on-site for IFS examination during surgery. Besides, we should investigate equipment renewal and additional imaging education in China and strengthen the popularization of sentinel node biopsy.

## Supplementary Information

**Additional file 1: Supplementary Table 1.** Cases selection in QUADAS-2.

**Additional file 2.** Supplementary materials.

## Data Availability

The datasets and images used during the current study are available from the corresponding author on reasonable request.

## Abbreviations

MI: Myometrial invasion
IGE: Intraoperative gross examination
IFS: Intraoperative frozen section
PS: Paraffin section
