# Supplementary material for: Preoperative and intraoperative assessment of myometrial invasion in patients with FIGO stage I non-endometrioid endometrial carcinoma—a large-scale, multi-center, and retrospective study
Source: Diagn Pathol. 2023 Jan 25;18:8. doi: 10.1186/s13000-023-01294-z (PMC9878924; doi:10.1186/s13000-023-01294-z)
Supplement: Supplementary file 1 — Additional file 1: Supplementary Table 1. Cases selection in QUADAS-2. [file 13000_2023_1294_MOESM1_ESM.docx]

Supplementary Table 1 Cases selection in QUADAS-2.

| **Cases Selection** |  |
| --- | --- |
| Whether the medical charts contain easily confused disease cases? | No |
| Whether the selection criteria of research objects are clear? | Yes |
| Whether continuous or random cases are included? | Yes |
| Whether the case-control design is avoided? | Yes |
| Whether inappropriate exclusions are avoided? | Yes |

Footnote QUADAS-2: Revised Tool for the Quality Assessment of Diagnostic Accuracy Studies.
